# Future access and improvement of industrial lactic acid bacteria cultures

**DOI:** 10.1186/s12934-017-0851-1

**Published:** 2017-12-21

**Authors:** Eric Johansen

**Affiliations:** Chr Hansen A/S, 10-12 Bøge Allé, Dk2970 Hørsholm, Denmark

**Keywords:** Fermentation, Biodiversity, Evolution, Nagoya Protocol, Biotechnology, Mutagenesis

## Abstract

Industrial fermentations based on micro-organisms such as the lactic acid bacteria (LAB) play an important role in several industries globally and represent multi-billion Euro/dollar businesses. LAB provide a natural way to produce safe, sustainable, and environmentally friendly products for a variety of industries. Product innovation is a key requirement for these industries to survive and grow globally. However, the development of new products may be affected by two man-made constraints; the Nagoya Protocol on benefit sharing and the opposition to the use of modern biotechnology for strain improvement. An expert workshop was held in Amsterdam, May 10–11, 2017 to discuss these challenges; a number of conclusions and recommendations were formulated and will be presented herein.

## Background

Fermentation has been used for millennia for the preservation of food and as a means to improve its organoleptic properties. Just as our ancestors domesticated plants and animals to provide a more stable food supply, the bacteria used in food fermentations can be considered to be domesticated. By selecting and propagating those organisms with desirable properties and excluding those with undesirable properties, mankind has fostered an evolution which ultimately led to a safer and more reliable food supply. However, the world is rapidly changing and evolution may be too slow to keep pace with the increased need for nutritious, safe, diverse and appealing food for an ever growing human population, especially in the context of climate change challenges.

One key component of evolution is genetic variation as the basis of selection. Variation can either be found in nature by screening, or introduced by scientists/breeders using a variety of established techniques [1]. Use of either of these approaches is currently being hampered by manmade constraints. Two specific issues are the restrictions on the use of modern biotechnology for strain improvement and the restrictions framed by the Convention on Biological Diversity (CBD) further detailed in the Nagoya Protocol [2]. These constraints were the subject of a 2-day expert workshop held in Amsterdam, May 10 and 11, 2017; the conclusions and recommendations from the workshop are presented herein.

As described on the CBD website [2]: “the objectives of the CBD are the conservation of biological diversity, the sustainable use of its components, and the fair and equitable sharing of the benefits arising from commercial and other utilization of genetic resources. The agreement covers all ecosystems, species, and genetic resources”. The Nagoya Protocol [2] stipulates that the benefits arising from the use of genetic resources shall be shared in a fair and equitable way. The intentions of the CBD and the Nagoya Protocol are considered valid and are fully supported by the workshop participants. However, as will be described below, there are a number of uncertainties about the interpretation of this protocol. The challenge is to ensure benefit sharing without removing the incentive for industrial use of the genetic resources; otherwise, there will be no benefit to share. In addition, academic research has always been dependent on the free sharing of research materials and restrictions on this would not be beneficial.

A number of techniques are available to induce changes in the genetic material of an organism. Some techniques such as mutation breeding have been used for decades and have remained uncontroversial while others, specifically those based on the use of recombinant DNA technology, have resulted in an intense debate [3]. Stringent regulations on the use of recombinant DNA technology in food and feed production have been developed in some parts of the world. A consequence of this is that companies that develop starter cultures for food fermentations are reluctant to introduce genetically modified organisms (GMOs) to the market [1]. Advanced technologies such as genome editing have not yet been fully integrated into the existing regulatory framework and their regulatory status in relation to the spectrum of techniques available to improve a trait of interest remains to be clarified. Regulatory uncertainty does not promote innovation.

Many of the bacteria used in food fermentation belong to the group known as lactic acid bacteria (LAB). These are used in the production of a large variety of fermented foods including cheese, yoghurt, sauerkraut, pickles, sausages, as well as in the production of animal feed (silage). Industrial production of these products has been based on the use of commercially produced starter cultures for more than a century.

The Lactic Acid Bacteria Industrial Platform (LABIP) is the industry platform for European Union-sponsored research programs on LAB [4]. LABIP is a European Economical Association, founded in 1994. The members of LABIP are companies that produce or use LAB and have production or research facilities within the EU. One of the aims of LABIP is to coordinate communication about topics of industrial relevance between academia, industry and EU authorities. LABIP was organizer and sponsor of the expert workshop “future access and improvement of industrial LAB cultures”.

## The Nagoya Protocol

The Nagoya Protocol is a supplementary agreement to the CBD. Its aim is to provide a transparent legal framework for the fair and equitable sharing of benefits arising from the utilization of biological resources, thereby contributing to the conservation and sustainable use of biodiversity. The Nagoya Protocol was adopted on the 29th of October, 2010 in Nagoya, Japan, and entered into force on October 12, 2014. There were more than 100 countries who were party to this Protocol at the end of 2017. The overall concept of the Nagoya Protocol is that before starting any research and development work on biological resources, prior informed consent (PIC) by the ‘provider country’ is needed. This is to be done according to mutually agreed terms (MAT) to be laid down in a contract describing access to the materials and how benefits will be shared. Benefit sharing can take a variety of forms including monetary payments, for example royalties or research funding, but also non-monetary forms such as technology transfer or scientific collaborations.

Implementation of the Nagoya Protocol raises a number of questions. For example, a precise definition of ‘utilization’ is required to allow an unambiguous determination of when the conditions of the Nagoya Protocol are to be invoked. If these are invoked too early in a project, the regulatory burden may keep companies and academic researchers from exploring resources from (biodiversity-rich) provider countries. This is especially relevant in screening campaigns of large numbers of strains and biodiversity assessments. Another concern is the establishment of which country has sovereign rights over highly mobile genetic resources such as bacteria and bacteriophages, especially considering the tenet that ‘*Everything is everywhere*, but, *the environment selects*’ first formulated by the microbiologist Baas Becking in 1934 (discussed by de Wit and Bouvier [5]). Finally, there is the question of in silico descriptions of genetic materials which can be analyzed without access to the genetic resource itself but which form the basis of much of modern biotechnology. Defining ‘sovereign rights’ can be difficult, especially considering that gene sequences exist for organisms which became extinct far before any of the current national boundaries were established.

## LABIP position on the Nagoya Protocol

The intentions of both the CBD and the Nagoya Protocol are valid and fully supported by LABIP. However, the rules should encourage the use of genetic resources from provider countries, not restrict it. This calls for a pragmatic approach where the efforts required to obtain PIC and MAT are proportional to the value of the immediate foreseen benefits. Otherwise there will be no benefit to share. A number of specific recommendations were formulated:

1. Industry and academia should work together to share ‘best practice’ solutions.
2. Precise definitions of terms like utilization and research and development are required so there is regulatory certainty about what is included and what is excluded from the Nagoya Protocol; in addition, precise definitions are an important prerequisite for moving towards harmonized Access and Benefit Sharing (ABS) legislation among provider countries across the globe.
3. Screening of a large number of strains to find a few candidates with specific characteristics and biodiversity assessments should be excluded from the scope of the Nagoya Protocol and of national ABS legislation, as the regulatory burden is disproportionately high; research and product development activities on the few selected candidates from a screening campaign should remain in scope, as there is a reasonable chance of commercialization.
4. Research using digital sequence information should remain outside the scope of the Nagoya Protocol and national ABS legislation, as it would be a daunting task to obtain PIC and MAT for all relevant sequences in a database such as GenBank. Public sequence databases were created with the express ambition of openly sharing, accessing and using such data, for users in developed and developing countries alike.
5. The human microbiome should be specifically excluded because it is not considered ethical for any government to have sovereign rights to such an important element of human physiology. This also avoids questions of ownership and ‘nationality’ of a microbe as people travel around the world and the microbe moves from the human body into the environment.

## Mutagenesis in the development of new starter cultures

The use of recombinant DNA technology for the improvement of LAB has a long history, and strains which could potentially be used in the food industry have been available for decades. However, due to regulatory concerns and a perceived lack of public acceptance, none of these have been brought to market, and industry has focused on strain improvement methods that do not involve the use of recombinant DNA technology [1].

The primary tool used in strain development is the isolation of mutants with specific desired properties. When a strong selection pressure can be exerted, spontaneous mutants can be isolated while in other cases, the use of a mutagen is required. Gentle mutagenesis is normally done with ultraviolet light while chemical mutagenesis is used when harsher conditions are required. Regardless of the method chosen, in order to be efficient enough to allow isolation of mutants, mutagenesis will generate a number of second-site mutations in the genome. Consequently, it has been recommended that whole genome sequencing be applied to identify and help evaluate the consequences of these changes on the functionality and safety of the developed strains [6].

There is a preference in the industry for spontaneous mutants or the use of very precise techniques for introducing genetic changes. Recent developments in genome editing and especially the use of CRISPR/Cas systems have considerable potential in this regard [7]. Using these techniques, it is possible to specifically change or remove a single base pair at a predetermined site. It is also possible to precisely delete many base pairs, allowing the precise excision of sequences which may be considered undesirable in the food supply or which might interfere with the desired functionality of a strain. In addition, sequences can be substituted with material from other strains or species. If material from other species is introduced, the resulting strain will be transgenic while introduction of material from the same species will result in cisgenic organisms. The regulatory status of transgenic strains is quite clear; they will be regulated as GMOs. The regulatory status of cisgenic strains and strains with introduced gene deletions or single base pair changes is currently uncertain.

## LABIP position on the use of modern techniques of mutagenesis

LABIP advocates that regulations should be science-based, enforceable, proportionate, non-discriminatory and provide a legal certainty to allow the development of long-term solutions to providing nutritious food to the growing human population. Regulations should, thus, reflect risk and be based on the properties of developed strains, not the methods used to make them.

Identical/indistinguishable strains should be regulated identically regardless of how they are obtained; otherwise, any regulations become unenforceable [8]. This implies that some uses of genome editing should be considered identical to other, more traditional mutagenesis techniques. As an interesting example consider: Selle et al. [9] used a selection scheme based on CRISPR/Cas9 to obtain strains of *Streptococcus thermophilus* in which the *prtS* gene and flanking regions have been deleted; while Bassi et al. [10] used adaptive laboratory evolution to isolate identical mutants. In both cases, the deletion event itself was spontaneous, occurring by homologous recombination between two insertion sequences flanking the *prtS* gene. Similarly, genome editing can be used to create strains which are identical to spontaneous mutants. Whole genome sequencing would be unable to determine which strain was obtained by which method and so they must present identical risks. Custers [8] has analysed the EU regulations concerning GMOs and concluded that organisms with genetic alterations that can also occur in nature are outside the scope of the GMO definition and so should not be more stringently regulated than organisms developed using traditional methods. LABIP supports this conclusion.

## Conclusions

The overall ambition of the participants of the LABIP Workshop “Future access and improvement of industrial LAB cultures” is to develop bacterial strains for food fermentations which are best suited to help nourish the ever growing human population by providing safe, nutritious and enjoyable food while minimizing the impact on the environment. Constraints which delay this development include regulatory uncertainties surrounding the Nagoya Protocol and limitations on the use of new techniques such as genome editing for creating strains with precisely defined, predetermined genetic variations.

New strains with specific traits can be obtained from nature, from traditional fermented foods, or from other manmade sources. There is no doubt that when traditional fermented foods are used as the source of superior commercial strains, benefits should be shared with the rightful owners of the technology. The main concern discussed at the workshop is that the current interpretation of the Nagoya Protocol appears to be so burdensome that it is tempting to source strains from countries which do not exert their sovereign rights and consequently would not require benefit sharing. Moreover, the Nagoya Protocol could also hinder research on natural bacterial biodiversity in countries exerting their sovereign right. This is the exact opposite effect of the intentions of the CBD and the Nagoya Protocol but is feasible due to the wide geographical distribution of most bacteria. A number of recommendations could ease this regulatory burden. For example, defining that screening of a large number of candidate strains is not utilization would have a major impact. The vast majority of the strains being studied will never become part of a commercial product and consequently, there would be no tangible benefits, and efforts to define PIC and MAT on these would be of no value. Once screening has identified the most promising candidates, bringing these to market would still be considered to be utilization and real benefits which can be shared would accrue.

Genome editing is more precise and consequently less likely to have unintended consequences than many traditional methods of strain improvement. It would be unfortunate if all uses of genome editing were combined into one category and defined to be GMOs. Instead, a case-by-case evaluation should be done, and strains obtained by genome editing, which could also be reasonably obtained by techniques such as conjugation, mutagenesis or other classical strain improvement methods, should be subject to the same regulatory burden and treatment as such strains.

It is hoped that the perspectives put forth here lead to an increased dialogue on these topics and ultimately result in a pragmatic approach to benefit sharing as well as the use of the most precise techniques for the improvement of bacterial strains for use in food fermentation.

## Abbreviations

ABS: access and benefit sharing
CBD: Convention on Biological Diversity
GMOs: genetically modified organisms
LAB: lactic acid bacteria
LABIP: Lactic Acid Bacteria Industrial Platform
MAT: mutually agreed terms
PIC: prior informed consent
